# A Clinical Cut-Off Value for the Systemic Immune-Inflammation Index to Predict Frequent Exacerbations in Stable COPD

**DOI:** 10.3390/medicina62030429

**Published:** 2026-02-24

**Authors:** Ozlem Sengoren Dikis, Ceren Degirmenci, Sabri Serhan Olcay, Fulden Cantas Turkis, Hacer Aybike Toptas Ogut, Utku Tapan, Fatih Alasan, Ozge Oral Tapan

**Affiliations:** 1 Department of Chest Diseases, Faculty of Medicine, Muğla Sıtkı Koçman University, Muğla 48000, Türkiye; ozlemsengoren@mu.edu.tr (O.S.D.); aybike125@gmail.com (H.A.T.O.); utkutapan@mu.edu.tr (U.T.); fatihalasan@mu.edu.tr (F.A.); ozgeoral@mu.edu.tr (O.O.T.); 2 Department of Chest Diseases, Muğla Research and Training Hospital, Muğla 48000, Türkiye; cerencekeli@hotmail.com; 3 Department of Biostatistics, Faculty of Medicine, Muğla Sıtkı Koçman University, Muğla 48000, Türkiye; fuldencantas@mu.edu.tr

**Keywords:** stable COPD, acute exacerbation, chronic inflammation, systemic immune-inflammation index (SII), risk marker

## Abstract

*Objective*: Acute exacerbations (AECOPD) are primary determinants of clinical instability in chronic obstructive pulmonary disease (COPD), and the “frequent exacerbator” (≥2/year) phenotype markedly increases morbidity and healthcare utilization. In this study, we evaluated the association between the Systemic Immune-Inflammation Index (SII), calculated from routine hemogram parameters during the stable period, and the occurrence of frequent exacerbations within the subsequent 1 year, and aimed to define a clinically applicable SII threshold (cut-off). *Materials and Methods*: In this retrospective observational cohort study conducted at a tertiary care center, patients who attended the outpatient clinic between January 2020 and February 2025 and had COPD confirmed by post-bronchodilator spirometric criteria (FEV_1_/FVC < 70%) were identified through electronic medical records. The index date was defined as a routine outpatient visit during stable COPD; patients were followed for AECOPD for 365 days after the index date. The stable period was defined as a visit occurring ≥4 weeks after the last exacerbation and without signs of acute infection. Patients with positive COVID-19 PCR results were excluded due to the uncertainty in distinguishing exacerbation from COVID-19. The primary endpoint was the development of frequent exacerbations (≥2 AECOPD) within 365 days. AECOPD was defined as an acute worsening of dyspnea, cough, and/or sputum requiring additional pharmacotherapy (systemic corticosteroids and/or antibiotics). SII, NLR, PLR, LMR, and PPN were calculated using hemogram parameters. Groups (<2 vs. ≥2 exacerbations) were compared; a ROC–Youden analysis was performed to determine cut-offs. After ROC-based dichotomization, univariate and multivariable logistic regression analyses were used to evaluate associations; multicollinearity was assessed using the VIF. To address potential optimism bias, diagnostic performance metrics (AUC, sensitivity, specificity) were internally validated using 1000 stratified bootstrap replicates. *Results*: A total of 159 patients were included. The cohort was predominantly male (91.2%). Demographic characteristics and most spirometric parameters were similar between groups; a trend toward lower absolute FVC was observed in the ≥2 exacerbation group (*p* = 0.051). Platelet counts were higher in the ≥2 exacerbation group (*p* = 0.029). In the ROC analysis, AUC values ranged from 0.505 to 0.591 across indices. For the SII, the AUC was 0.591 (95% CI: 0.500–0.677; *p* = 0.049), and the optimal cut-off was 1082.79. The LMR cut-off was 1.76; however, the LMR did not demonstrate statistically significant discriminatory performance in the ROC analysis (AUC 0.535; *p* = 0.448). In univariate analyses, SII > 1082.79 (OR = 3.028, 95% CI: 1.522–6.027; *p* = 0.002) was associated with frequent exacerbations. In a multivariable logistic regression adjusted for cardiovascular disease and overall comorbidity status, SII > 1082.79 remained independently associated (OR = 3.029, 95% CI: 1.485–6.179; *p* = 0.002). Other hemogram-derived indices did not retain independent prognostic significance in this outpatient cohort. *Conclusion*: SII measured during stable COPD was independently associated with frequent exacerbations over the subsequent 1 year. The SII > 1082.79 threshold may offer a practical risk stratification approach to flag “high-risk” patients in outpatient care. However, given the modest discriminative performance and the single-cohort derivation, this cut-off should be considered exploratory despite the use of bootstrap internal validation. Because this was a single-center study with a predominantly male cohort, the generalizability—particularly to female patients and other settings—requires prospective external validation.

## 1. Introduction

Chronic Obstructive Pulmonary Disease (COPD) is a heterogeneous lung disease that arises from chronic exposure to noxious particles and gases; it is accompanied by persistent inflammation and immune dysregulation in the airway and alveolar compartments, and leads—through structural remodeling—to airflow limitation that is limited in reversibility and generally progressive [1]. In COPD, an increased proinflammatory cytokine response and oxidative stress sustain the chronic inflammatory microenvironment and drive disease progression [2,3]. At the endotype level, particularly in subgroups where neutrophilic inflammation predominates, epithelial activation, protease–antiprotease imbalance, and immune activation constitute the pathobiological basis of small airway injury and parenchymal destruction [3]. Along this axis, lung macrophages, as long-lived effector cells, both respond to environmental stimuli and produce mediators that regulate the behavior of surrounding cells; they also play a critical role in the clearance of apoptotic neutrophils, and it is thought that increased macrophage burden and enhanced protease potential in COPD may accelerate tissue injury [4]. In parallel, a Th1/Th17-predominant inflammatory milieu may strengthen epithelial proinflammatory responses to infectious triggers and modulate the innate/adaptive components of the immune response, thereby providing a mechanistic framework underlying clinical instability in COPD [5].

Acute exacerbations of COPD (AECOPD) are events characterized by acute inflammatory amplification superimposed on the chronic inflammatory burden and have a marked effect on the clinical course [1]. An exacerbation manifests as a rapid, pronounced worsening of symptoms such as increased dyspnea, cough, and/or sputum production, and is triggered in most cases by infections, environmental pollutants, or other inflammatory stimuli [1]. During this process, the intensification of airway inflammation-particularly increased epithelial activation and the neutrophilic response-forms the biological basis of clinical deterioration [3,5]. Exacerbations reduce quality of life, increase hospitalization and readmission rates, and accelerate lung function decline, thereby adversely affecting prognosis [1]. Because of these adverse clinical and economic consequences, early identification of patients with a high exacerbation burden is critical for targeting preventive strategies; however, the multifactorial nature of COPD pathogenesis-including heterogeneity in environmental exposures and genetic components-makes the clinical prediction of this risk difficult [6].

This difficulty in clinical prediction has increased interest in accessible and cost-effective biomarkers that can capture systemic inflammation that may persisting even during the stable phase. In this context, composite indices derived from complete blood count parameters-because they jointly reflect the balance between systemic inflammatory activation and immune competence-have emerged as attractive candidates. The Systemic Immune-Inflammation Index (SII), which combines neutrophil, lymphocyte, and platelet counts, is a composite marker the reflecting systemic inflammation–immune balance [7]. Biologically, a high SII indicates a proinflammatory and relatively immunosuppressed profile characterized by increased neutrophils and platelets accompanied by decreased lymphocytes [7,8]. Although data in the COPD context are limited, population-based studies have reported that the SII may be associated with COPD risk, severity, and lung function, and higher SII levels may be linked to poor clinical outcomes [9,10,11]. However, in stable COPD, most existing evidence on the prognostic role of the SII has either been limited by a cross-sectional design or focused on composite outcomes such as mortality or hospitalization; its capacity to predict future exacerbations—particularly frequent exacerbations—that directly affect clinical management has not been adequately evaluated. Moreover, a standardized and validated threshold (cut-off) defining a “high-risk” group, which is a prerequisite for translating the prognostic information of the SII into clinical action, is not yet available. These critical gaps represent one of the main barriers limiting the adoption of the SII as a routine decision-support tool in outpatient practice.

In this study, through a retrospective analysis of prospectively recorded follow-up data from stable COPD patients attending our outpatient clinic, we tested the hypothesis that higher baseline SII levels during the stable period would be associated with the development of frequent exacerbations (≥2/year) within the subsequent 1 year, and that a quantitative SII threshold could contribute to risk stratification in the outpatient setting. The primary objective was to evaluate the association between the SII calculated in the stable period and the development of frequent exacerbations over the following 1 year. Secondary objectives were to [1] define a quantitative SII threshold (cut-off) applicable in clinical practice, [2] evaluate the performance of this cut-off and continuous SII in discriminating frequent exacerbation risk (e.g., AUC, sensitivity, specificity), and [3] examine the prognostic value of the SII together with or in comparison to established clinical risk factors.

## 2. Materials and Methods

### 2.1. Study Design and Patient Population

This was a retrospective observational cohort study conducted at a tertiary care center. Patients who attended the outpatient clinic between January 2020 and February 2025 and had COPD confirmed by according to post-bronchodilator spirometric criteria (FEV_1_/FVC < 70%) were identified through electronic medical records.

For each patient, the index date was defined as a routine outpatient visit during a period of stable COPD. Clinical and laboratory data obtained on this date were considered baseline measurements. Patients were followed longitudinally through medical records for 365 days after the index date and evaluated for acute COPD exacerbations (AECOPD).

Inclusion criteria were:

(i) age ≥18 years,
(ii) spirometry-confirmed COPD diagnosis, and
(iii) complete 1-year follow-up data after the index date.

**Figure 1:**
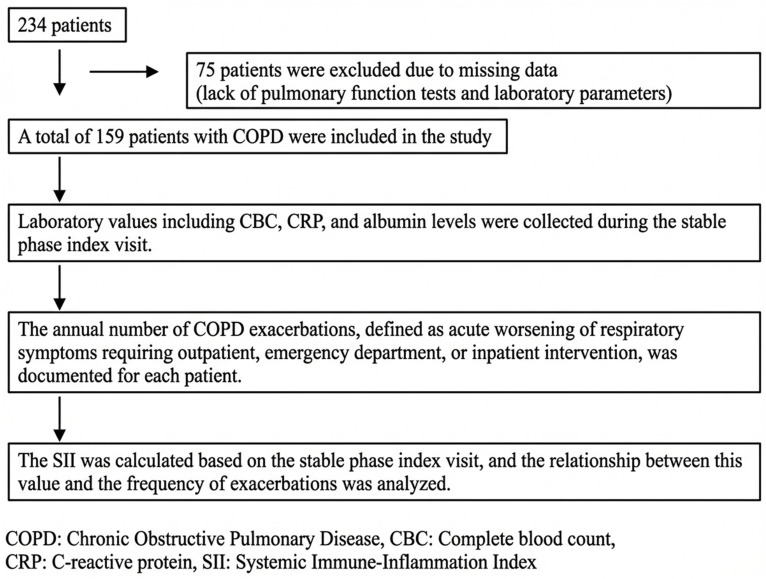
Study Flow Diagram.

A total of 159 patients meeting these criteria were included in the analyses.Patients with a positive COVID-19 PCR test were excluded. The clinical presentation in these cases could not be reliably distinguished between COPD exacerbation and COVID-19 infection/pneumonia; therefore, patients with COVID-19 PCR positivity were not included in the analyses (Figure 1).

### 2.2. Definition of Stable COPD Period

The “stable period” was defined as at least 4 weeks after the last AECOPD episode, with no signs of acute respiratory tract infection (fever, increased purulent sputum, acute increase in dyspnea) and presenting for routine outpatient follow-up. Complete blood count values for the stable period were obtained at this visit or within a maximum of 14 days prior to the visit, from measurements without accompanying evidence of acute infection.

### 2.3. Data Collection

Demographic and clinical data, including age, sex, height, weight, and comorbidities (hypertension, diabetes mellitus, coronary artery disease, and malignancy) were obtained from the electronic medical record system. The recorded spirometric parameters were FEV_1_, FVC, and the FEV_1_/FVC ratio. Laboratory data including complete blood count (CBC), C-reactive protein (CRP), and albumin levels were recorded both during the stable period and during visits related to exacerbations.

### 2.4. Outcome Definitions

The primary outcome was defined as frequent exacerbations developing within 365 days after the index date, classified as ≥2 AECOPD per year.

AECOPD was defined as an acute worsening of dyspnea, cough, and/or sputum volume or purulence compared with baseline symptoms that required additional pharmacologic therapy (systemic corticosteroids and/or antibiotics). Exacerbation episodes were verified through outpatient visit records, emergency department records, and hospitalization records.

### 2.5. Calculation of Inflammatory Indices

The following indices were calculated using complete blood count parameters:

$$SII=\frac{Platelet\;count*Neutrophil\;count}{Lymphocyte\;count}\;NLR=\frac{Neutrophil\;count}{Lymphocyte\;count}\;PLR=\frac{Platelet\;count}{Lymphocyte\;count}\;PPN=Platelet\;count*Neutrophil\;LMR=\frac{Lymphocyte\;count}{Monocyte\;count}$$

### 2.6. Statistical Analysis

All statistical analyses were performed using RStudio (version 2025.09.0). Distributional characteristics of continuous variables were evaluated using visual inspection and summary statistics. Normally distributed continuous variables are presented as mean ± standard deviation, whereas non-normally distributed variables are presented as median (minimum–maximum). Categorical variables are expressed as frequencies and percentages. Baseline characteristics were compared according to exacerbation frequency (<2 vs. ≥2 exacerbations). Categorical variables were compared using the Pearson’s chi-square test or Fisher’s exact test, as appropriate. Continuous variables were compared using the independent samples t-test for normally distributed data and the Mann–Whitney U test for non-normally distributed data.

Receiver operating characteristic (ROC) curve analysis was performed to derive operational cut-off values for inflammatory indices including LMR, NLR, PLR, PPN, and SII. Optimal cut-off points were determined using the Youden index. To address potential optimism bias and ensure internal validation, 95% confidence intervals (CI) for AUC, sensitivity, and specificity were calculated using 1000 stratified bootstrap replicates. Following dichotomization based on ROC-derived cut-off values, univariate binary logistic regression analyses were performed to examine associations between inflammatory indices and frequent exacerbations. Odds ratios (ORs) and corresponding 95% CIs were reported.

Variables that were statistically significant at *p* < 0.10 in univariate analyses were subsequently included in a multivariable logistic regression model to identify factors independently associated with frequent exacerbations. Due to the retrospective nature of the database, prior-year exacerbation history and inhaled corticosteroid (ICS) therapy profiles were not available for systematic inclusion as covariates; thus, the model focused on available clinical comorbidities and significant inflammatory markers. Multicollinearity among covariates was assessed using the variance inflation factor (VIF), with values <5 considered acceptable. Model calibration and fit were evaluated using the Hosmer–Lemeshow goodness-of-fit test and the omnibus test. All statistical tests were two-sided, and *p* < 0.05 was considered statistically significant.

## 3. Results

Table 1 summarizes baseline clinical, laboratory, and inflammatory characteristics of the study population stratified by exacerbation frequency. To ensure that inflammatory indices were evaluated in the absence of acute infection, CRP and albumin levels were compared between groups and no significant difference was detected; this finding indicates that inflammatory status at the time of sampling was comparable between groups. Similarly, leukocyte subsets associated with acute inflammatory response—including neutrophil and lymphocyte counts—were comparable between groups.

Demographic characteristics and pulmonary function parameters were generally similar across exacerbation categories. Absolute FVC values showed a non-significant trend toward lower levels in patients with ≥2 exacerbations (*p* = 0.051), while other spirometric measures did not differ significantly. Platelet counts were higher in the ≥2 exacerbation group (*p* = 0.029).

ROC curve analysis was performed to determine the optimal cut-off values for the LMR, NLR, PLR, PPN, and SII indices in relation to frequent exacerbations. To account for potential optimism bias and ensure internal validation, 1000 stratified bootstrap replicates were employed to calculate 95% confidence intervals (CI) for all diagnostic metrics (Table 2). Among the evaluated indices, AUC values ranged from 0.505 to 0.591. The SII demonstrated the highest diagnostic performance with an AUC of 0.591 (95% CI: 0.500–0.677; *p* = 0.049). At the optimal cut-off of >1082.79, the SII yielded a sensitivity of 45.83% (95% CI: 30.56–73.65) and a specificity of 78.16% (95% CI: 47.10–89.66). In contrast, the AUC values for LMR, NLR, PLR, and PPN were lower and did not reach statistical significance (Table 2). 

In univariate logistic regression analyses, a higher systemic immune-inflammation index (SII > 1082.79) was significantly associated with frequent exacerbations, with approximately three-fold higher odds (OR: 3.028, 95% CI: 1.522–6.027; *p* = 0.002). Cardiovascular disease (OR: 0.430, 95% CI: 0.224–0.827; *p* = 0.011) and the presence of comorbidity (OR: 0.426, 95% CI: 0.221–0.821; *p* = 0.011) were also significantly associated with exacerbation frequency in univariate analyses. No statistically significant associations were observed for diabetes mellitus, chronic kidney disease, hypothyroidism, solid organ malignancy, hematological malignancy, or autoimmune disease (all *p* > 0.05) (Table 3).

In the multivariable logistic regression model, after adjustment for cardiovascular disease and comorbidity status, SII > 1082.79 remained independently associated with frequent exacerbations (OR: 3.029, 95% CI: 1.485–6.179; *p* = 0.002). The associations observed for cardiovascular disease and comorbidity in univariate analyses were reduced and did not remain statistically significant in the adjusted model. Variance inflation factor (VIF) values ranged from 1.021 to 1.691, indicating no evidence of relevant multicollinearity among the variables included in the model (Table 3).

## 4. Discussion

Our study demonstrates that higher SII values measured during the stable period are associated with frequent exacerbations in COPD and proposes a quantitative risk stratification threshold (SII > 1082.79) that may be usable in outpatient practice. In our study, comparing CRP and albumin levels between groups and finding no significant difference—aimed at reducing the likelihood of acute infection—suggests that systemic inflammatory status at the time of sampling was similar and that differences observed in hemogram-derived indices may be related to stable-phase inflammatory burden rather than an acute-phase response due to acute infection. This finding indicates that the observed association may be linked to inflammatory activity during the stable phase rather than an “acute-phase response”. Indeed, our results support the concept that persistent low-grade systemic inflammation is associated with disease instability and the prediction of exacerbations in COPD [12,13].

In COPD, acute exacerbations are not only episodes that accelerate disease progression; they are key clinical outcomes that define the “frequent exacerbator” phenotype, which tends to recur partially independently of disease severity and significantly affects prognosis [14]. It has been shown that systemic inflammation that may persist even during the stable period plays an important role in the biological basis of this phenotype, and that peripheral blood biomarkers in some cohorts may be associated with future exacerbation burden [15,16]. In this framework, our study evaluates the potential of hemogram-derived inflammatory indices in stable COPD patients to predict the risk of frequent exacerbations (≥2/year) over the next 1 year in an outpatient setting and provides a testable threshold approach for the SII.

Our findings for SII provide two clinical implications: first, SII levels are associated with the risk of frequent exacerbations; second, the 1082.79 cut-off identified by ROC analysis—given its relatively high specificity (78.16%)—appears to be a candidate operational threshold particularly for flagging a “high-risk” subgroup. However, the AUC was statistically significant but had limited discriminative power (0.591) and the modest sensitivity level requires positioning SII not as a stand-alone decisive test, but as a risk marker that may contribute more meaningfully when used together with strong clinical determinants (especially prior exacerbation history, symptom burden, treatment profile, and comorbidities). Therefore, the cut-off value proposed by our study is not a “final boundary”, but should be considered a quantitative initial reference that can be standardized through further validation studies for a biomarker with high applicability in the outpatient setting. In our multivariate model, the SII remained independently associated with frequent exacerbations after adjusting for cardiovascular disease and overall comorbidity status. However, the term ‘independent’ should be interpreted with caution as our model could not account for prior exacerbation history and ICS use-two of the strongest predictors in COPD-due to incomplete electronic records. Consequently, the SII serves as a complementary adjunct that provides biological context where clinical history may be insufficient.

A possible biological explanation for SII producing a more pronounced signal than the NLR or PLR in some cohorts may be that it reflects three distinct cellular components simultaneously. Systemic inflammation in COPD involves not only neutrophilic activation but also changes in the lymphocyte pool representing adaptive immune response and the participation of platelets in inflammatory processes; while SII captures this triple axis (neutrophil–lymphocyte–platelet) together, the NLR and PLR reflect more “binary” interactions. Therefore, the SII may represent a more holistic marker of immune dysfunction for a multifactorial and complex clinical outcome such as frequent exacerbations; the independent association of the SII in our findings is consistent with this hypothesis [12,17].

When evaluating the literature on the SII, its association with stable-phase clinical severity indicators is not consistent across studies. In the study by Celtikci et al., no significant relationship was shown between the SII and FEV_1_, smoking status, or symptom burden in stable COPD patients; this was interpreted as a finding that may be related to cohort characteristics and follow-up duration [17]. In contrast, there are studies supporting an association between SII and more severe clinical presentations and poor short-term outcomes: Zhang et al. found higher SII levels to be associated with respiratory failure and in-hospital mortality [11], and Giri et al. reported that the SII independently predicted 30-day mortality in critically ill COPD patients [18]. In addition, retrospective data comparing stable and exacerbation phases showed that the SII increases during exacerbations and is associated with the clinical course [19]. Within this body of evidence, our demonstration of an association between stable-phase SII and frequent exacerbations over the next 1 year supports the possibility that the SII may be linked not only to hospital outcomes but also to exacerbation burden representing clinical instability that is critical for outpatient follow-up.

Regarding hemogram-derived indices other than the SII, the literature is more heterogeneous. For the NLR, some large cohorts have reported that elevated stable-phase NLR may be associated with future exacerbations and mortality [20,21]. In contrast, the lack of significance for the NLR in our cohort may be explained by factors such as sample size, differences in cut-offs, heterogeneity of exacerbation etiologies during follow-up, treatment profile (especially ICS use), and a sharper outcome definition such as “≥2/year”, which may limit the discriminative power of indices. For the PLR, although studies show that it rises particularly during acute exacerbations and may be associated with poor short-term outcomes [22,23], an independent contribution to predicting future frequent exacerbations in the stable phase may not be replicated in every cohort. Indeed, in the TIE cohort, while stable-phase CRP, fibrinogen, and WBC retained independent associations with future AECOPD frequency, composite hemogram indices such as the PLR, SII, SIRI, and AISI did not provide additional independent prognostic contribution in fully adjusted models [24]. This suggests that the prognostic performance of composite indices may be sensitive to cohort selection, exacerbation definition, follow-up duration, and strong clinical determinants included in the model (e.g., prior exacerbation history); therefore, our findings should be interpreted as a complementary contribution showing that the SII may produce an additional risk stratification signal in a specific outpatient context.

Our LMR findings are noteworthy in emphasizing the distinction between the “screening” and “confirmatory” uses of indices. In the ROC analysis, the high sensitivity and low specificity of the LMR suggest that it may have an “early warning/screening” character to avoid missing high-risk patients, but should not be treated as a confirmatory threshold on its own. On the other hand, the persistence of an independent association in logistic regression analyses suggests that the monocyte–lymphocyte balance may carry a meaningful biological signal within the axis of chronic inflammatory activation and immune response impairment in COPD. Evidence in this area is evolving, and systematic evaluations emphasize that different biomarkers may show variable performance across outcomes and highlight the need for validation [25,26]. For PPN, given the limited standardized indications and validated thresholds in COPD, it would be more prudent to consider this index as an exploratory candidate at this stage.

The clinical output of our study can be concretized within a simple decision-support approach: identification of an SII > 1082.79 in a stable COPD patient may be considered a “high-risk marker” that triggers reviewing treatment adherence and inhaler technique, closer management of comorbidities (especially cardiovascular disease), and optimization of vaccination status [27]. This approach avoids using the SII as a stand-alone diagnostic test while helping allocate clinical resources toward patients at higher risk.

It is important to note that the SII cut-off value of 1082.79 identified in this study should be considered exploratory and hypothesis-generating due to the derivation within a single cohort. Given the modest AUC of 0.591, the SII is best utilized as a complementary clinical adjunct rather than a stand-alone predictive tool, providing additional inflammatory context alongside established risk factors.

Although this study contributes to the growing body of evidence, it has several limitations. The retrospective and single-center design limits generalizability; therefore, the threshold we propose should be tested in prospective cohorts across different centers and clinical phenotype distributions. Moreover, the study population was predominantly male, reflecting the sex distribution of our outpatient cohort. While this does not invalidate the observed associations, it may limit the applicability of the proposed SII threshold to female patients and to populations with different sex distributions. Accordingly, clinical implications should be interpreted cautiously until externally validated in larger, multicenter cohorts with broader and more balanced representation. Crucially, due to the retrospective nature of our institutional database, certain key clinical parameters, such as prior exacerbation history and the exact dosage of inhaled corticosteroid (ICS) use, were not systematically recorded for the entire cohort and thus could not be included in the multivariable models. Furthermore, using ROC-derived cut-offs generated in the same dataset for association analyses may introduce optimism bias and potential overestimation of performance; however, we addressed this by performing internal validation with 1000 stratified bootstrap replicates to provide more robust and reliable estimates. Given these data-related constraints, the SII threshold of 1082.79 should be interpreted as strictly exploratory and hypothesis-generating. Despite these limitations, our findings provide a testable initial threshold approach for future multicenter and prospective studies.

## 5. Conclusions

Inflammatory indices easily obtained from routine complete blood counts in stable COPD patients—particularly SII—can be integrated into clinical decision support as practical and cost-effective adjunct tools for predicting exacerbation risk during follow-up. The SII > 1082.79 threshold proposed in our study, which was internally validated using 1000 stratified bootstrap replicates, provides a stable point of reference for identifying a “high-risk” subgroup with high specificity. However, given the retrospective design and the absence of certain systematically recorded clinical parameters such as prior exacerbation history and ICS dosage, this threshold should be strictly considered exploratory and hypothesis-generating. The modest discriminative power observed underscores that SII should function as a complementary marker alongside established clinical risk factors rather than a stand-alone diagnostic tool. The clinical utility of SII and other hemogram-derived indices should be clarified through prospective external validation and standardization in larger populations and in different clinical scenarios (e.g., stratification by ICS use).

## Figures and Tables

**Table 1:** Baseline demographic characteristics, comorbidities, pulmonary function parameters, hematological indices, and inflammatory markers of the study population stratified by exacerbation frequency.

	Overall (*n* = 159)	Exacerbation Frequency		Test Statistics	*p*	ES
		<2 (*n* = 87)	≥2 (*n* = 72)			
Demographic						
Gender Male	145 (91.2)	79 (90.8)	66 (91.7)	0.001	>0.999	0.015
Age (years)	67.79 ± 10.13	67 (43–87)	70 (40–86)	−0.603	0.547	0.056
BMI (kg/m^2^)	26.07 ± 5.09	26.56 ± 5.18	25.48 ± 4.96	1.338	0.183	0.213
Comorbidity						
Cardiovascular disease	66 (41.5)	44 (50.6)	22 (30.6)	6.503	**0.011**	0.202
Diabetes mellitus	21 (41.5)	13 (14.9)	8 (11.1)	0.226	0.635	0.056
Chronic kidney disease	5 (3.1)	2 (2.3)	3 (4.2)	0.659	0.659	0.053
Hypothyroidism	3 (1.9)	2 (2.3)	1 (1.4)	1.000	>0.999	0.033
Solid organ malignancy	17 (10.7)	8 (9.2)	9 (12.5)	0.171	0.679	0.053
Hematological Malignancy	4 (2.5)	3 (3.4)	1 (1.4)	0.627	0.627	0.065
Autoimmune disease	7 (4.4)	4 (4.6)	3 (4.2)	1.000	>0.999	0.010
Cerebrovascular disease	4 (2.5)	4 (4.6)	0 (0)	0.127	0.127	0.146
Pulmonary Function Test						
FVC (%)	77.12 ± 20.09	79.03 ± 19.24	74.81 ± 20.98	0.157	0.187	0.211
FVC (l)	2.67 ± 0.85	2.76 (1.21–5.01)	56.25 (5.43–69.60)	−1.962	0.051	−0.181
FEV1/FVC	55.17 ± 11.06	58.40 (29.70–69)	56.25 (5.43–69.60)	−0.427	0.669	−0.039
CBC						
Lymphocyte	2.03 ± 1.59	1.83 (0.37–19.22)	1.87 (0.28–3.86)	−0.014	0.989	0.001
Platelets	258.93 ± 75.25	239 (110–444)	268.50 (117–527)	−2.180	**0.029**	0.201
Neutrophil	5.38 ± 2.32	5 (1.58–19.61)	5.01 (2.09–12.91)	−0.439	0.660	−0.041
Monocyte	0.60 (0.51–0.78)	0.60 (0.10–4.90)	0.62 (0.09–1.43)	−0.836	0.403	−0.077
Acute phase reactants						
C-reactive protein	2.76 (1.58–5.42)	2.76 (0.09–82.93)	2.80 (0.28–27.74)	−0.680	0.497	−0.063
Albumin	41.68 ± 5.38	43 (4.88–51.70)	42.75 (29–56.40)	−0.312	0.755	−0.029
Inflammatory indices						
SII	769.94 (16.31–27751.91)	727.46 (16.31–10423)	965.21 (221.67–27751.91)	−1.972	**0.049**	0.182
NLR	2.58 (0.18–28.20)	2.87 (0.18–28.20)	2.50 (0.91–22.32)	−0.107	0.915	−0.001
PLR	134.01 (5.72–885.71)	134.01 (5.72–540.54)	136.13 (61.66–885.71)	−0.995	0.320	0.092
PPN	1257.25 (216.46–6803.57)	1255.98 (216.46–5550.72)	1279.78 (375.57–6803.57)	−0.606	0.545	0.056
LMR	2.83 (0.22–32.58)	2.66 (0.22–32.58)	2.89 (1.08–19.56)	−0.758	0.449	0.070

Data are presented as mean ± standard deviation for normally distributed continuous variables, median (minimum–maximum) for non-normally distributed continuous variables, and number (percentage) for categorical variables. The Overall column summarizes the entire study population, while comparisons were conducted between patients with <2 and ≥2 exacerbations. Between-group comparisons were performed using the independent samples t-test or the Mann–Whitney U test for continuous variables, according to distributional assumptions, and the Pearson chi-square test or Fisher’s exact test for categorical variables, as appropriate. All statistical tests were two-sided, and a *p* value < 0.05 was considered statistically significant. Statistically significant *p*-values are presented in bold. Effect size (ES) estimates are provided to quantify the magnitude of between-group differences, using Cohen’s d for parametric continuous variables, rank-biserial correlation for non-parametric continuous variables, and Cramér’s V for categorical variables. Abbreviations: BMI, body mass index; FVC, forced vital capacity; FEV1, forced expiratory volume in 1 s; CBC, complete blood count; SII, systemic immune-inflammation index; NLR, neutrophil-to-lymphocyte ratio; PLR, platelet-to-lymphocyte ratio; PPN, platelet × neutrophil product; LMR, lymphocyte-to-monocyte ratio.

**Table 2:** Diagnostic performance and bootstrap-based internal validation of systemic inflammatory indices for predicting frequent exacerbations (≥2).

Variables	Cut-Off Point	Sensitivity (95% CI)	Specificity (95% CI)	AUC (95% CI)	*p*
LMR	>1.76	93.06 (20.83–98.61)	22.99 (14.94–93.10)	0.535 (0.446–0.630)	0.448
NLR	≤2.82	63.89 (44.41–91.79)	50.57 (19.48–70.11)	0.505 (0.415–0.601)	0.912
PLR	>165.20	37.50 (16.67–95.87)	73.56 (14.94–95.40)	0.546 (0.453–0.634)	0.321
PPN	>1085.42	72.22 (33.33–98.61)	40.23 (11.49–80.46)	0.528 (0.436–0.619)	0.544
SII	>1082.79	45.83 (30.56–73.65)	78.16 (47.10–89.66)	0.591 (0.500–0.677)	**0.049**

AUC: Area under the curve; CI: Confidence interval; LMR: Lymphocyte-to-monocyte ratio; NLR: Neutrophil-to-lymphocyte ratio; PLR: Platelet-to-lymphocyte ratio; PPN: Platelet-to-neutrophil ratio; SII: Systemic immune-inflammation index. 95% CI’s for sensitivity, specificity, and AUC were derived from 1000 stratified bootstrap replicates for internal validation to minimize the risk of optimism bias. *p* < 0.05 was considered statistically significant. Statistically significant *p*-values are presented in bold.

**Table 3:** Univariate and multivariable logistic regression analyses of factors associated with frequent exacerbations.

Variables	Univariate		Multivariable		
	OR (95% CI)	*p*	OR (95% CI)	*p*	VIF
SII (>1082.79)	3.028 (1.522–6.027)	**0.002**	3.029 (1.485–6.179)	**0.002**	1.021
Cardiovascular disease (Yes)	0.430 (0.224–0.827)	**0.011**	0.514 (0.217–1.219)	0.131	1.667
Comorbidity (Yes)	0.426 (0.221–0.821)	**0.011**	0.698 (0.295–1.651)	0.414	1.691
Diabetes mellitus (Yes)	0.712 (0.277–1.825)	0.479			
Chronic kidney disease (Yes)	1.848 (0.300–11.372)	0.508			
Hypothyroidism (Yes)	0.599 (0.678–6.739)	0.678			
Solid organ malignancy (Yes)	1.411 (0.515–3.867)	0.504			
Hematological Malignancy (Yes)	0.394 (0.040–3.875)	0.425			
Autoimmune disease (Yes)	0.902 (0.195–4.169)	0.895			

OR: odds ratio; CI: confidence interval; SII: systemic immune-inflammation index; VIF: variance inflation factor. Univariate and multivariable logistic regression analyses were performed to evaluate factors associated with frequent exacerbations. Variables with *p* < 0.10 in univariate analyses were entered into the multivariable model. Model calibration was assessed using the Hosmer–Lemeshow goodness-of-fit test (χ^2^ = 4.748, *p* = 0.314), and overall model significance was evaluated using the omnibus test (*p* < 0.001). VIF values indicated no evidence of multicollinearity among the variables included in the multivariable model. All tests were two-sided, with statistical significance defined as *p* < 0.05; statistically significant *p*-values are presented in bold.

## Data Availability

Data supporting the findings of this study are available from the corresponding author upon reasonable request. The data were not publicly available because of ethical and privacy restrictions related to the patient’s confidentiality.
